# Large-scale and controllable synthesis of metal-free nitrogen-doped carbon nanofibers and nanocoils over water-soluble Na_2_CO_3_

**DOI:** 10.1186/1556-276X-8-545

**Published:** 2013-12-27

**Authors:** Qian Ding, Xueyin Song, Xiujuan Yao, Xiaosi Qi, Chak-Tong Au, Wei Zhong, Youwei Du

**Affiliations:** 1 Nanjing National Laboratory of Microstructures, Nanjing University, Nanjing 210093, People’s Republic of China; 2 Jiangsu Provincial Laboratory for Nanotechnology, Nanjing University, Nanjing 210093, People’s Republic of China; 3 College of Science, Guizhou University, Guiyang, 550025, People’s Republic of China; 4 Chemistry Department, Hong Kong Baptist University, Hong Kong 852, People’s Republic of China

**Keywords:** Carbon materials, Chemical vapor deposition, Water-soluble, Nitrogen-doped

## Abstract

Using acetylene as carbon source, ammonia as nitrogen source, and Na_2_CO_3_ powder as catalyst, we synthesized nitrogen-doped carbon nanofibers (N-CNFs) and carbon nanocoils (N-CNCs) selectively at 450°C and 500°C, respectively. The water-soluble Na_2_CO_3_ is removed through simple washing with water and the nitrogen-doped carbon nanomaterials can be collected in high purity. The approach is simple, inexpensive, and environment-benign; it can be used for controlled production of N-CNFs or N-CNCs. We report the role of catalyst, the effect of pyrolysis temperature, and the photoluminescence properties of the as-harvested N-CNFs and N-CNCs.

## Background

Since Iijima’s paper on helical carbon nanotubes, carbon nanomaterials (CNM) such as carbon nanotubes (CNT) and carbon nanofibers (CNF) have attracted great attention for their unique and outstanding electrical and mechanical properties [1-4]. The helical CNT are composed of five-membered or seven-membered rings, having carbon atoms of *sp*^2^ and *sp*^3^ hybridization [5,6]. It is envisaged that helical CNT exhibit novel and peculiar properties that are different from those of linear CNT. It has been suggested that CNM can be utilized in hydrogen storage [7,8], microwave absorption [9], and field emission [10,11]. Using CNM, scientists tried to fabricate nanosized electromagnetism devices [12-14] such as solenoid switch [15,16], miniature antenna [17,18], energy converter [19,20], and sensor [21,22].

For CNM generation, methods such as arc discharge, laser ablation, hydrothermal carbonization, solvothermal reduction, and chemical vapor deposition (CVD) are used [23-28]. Nonetheless, it is common to have metal impurities in the products, and the intrinsic properties of the as-obtained CNM are uncertain. The problem of metal impurities hinders further researches on CNM especially those related to electromagnetism features [29,30]. It is tedious and costly to remove metal impurities such as those of iron-group elements or their alloys [31]. Furthermore, unexpected defects or contaminants could be introduced into the CNM during purification procedures.

As a traditional method, CVD has its advantages [32,33]. By regulating parameters such as catalyst amount, reaction temperature, source flow rate, one can obtain different kinds of CNM. It is possible to control the CVD process for a designated outcome by adopting a particular set of reaction conditions [34,35]. Using acetylene as carbon precursor, Amelinckx et al. [36], Nitze et al. [37], and Tang et al. [38] obtained CNM with high purity and selectivity. Nevertheless, there are disadvantages such as high reaction temperature and outgrowth of desired product [28,39]. As for the growth mechanism of CNT in CVD processes, there are still controversies [40,41].

By doping foreign elements such as nitrogen and boron into the graphite lattices of CNM, Wang et al. [42], Ayala et al. [43], and Koós et al. [44] induced crystal and electronic changes to the structures of CNM [42-44]. It is noted that as support for palladium nanoparticles, helical CNM show excellent properties in electro-catalytic applications [45,46]. According to Franceschini et al. [47] and Mandumpal et al. [48], the introduction of nitrogen restrains the aggregation of vacancies, resulting in defects and dislocations, as well as amplified curvature of graphite planes. The results of both experimental and theoretical studies demonstrate that compared to pure CNT, nitrogen-doped CNT show enhanced field emission properties and there is a shift of the dominant emission towards lower energies [49-51]. Through theoretical studies of heteroatom-substituted graphite systems, Hagiri et al. suggested that different heteroatom arrangements cause different spin-stable singlet and triplet states and that the substituted nitrogen atom as a spin cap induces the π electron excess [52]. When it comes to CNT utilization, high incorporation of nitrogen is desirable in promoting porosity and electrochemical reactivity of CNT. On the other hand, if CNT are supposed to be applied in semiconductor technology, low nitrogen-doping density is necessary.

Recently, we reported the large-scale synthesis of various kinds of non-doped CNM that are metal-free [53-55]. Herein, we report the use of Na_2_CO_3_ as catalyst for the selective formation of nitrogen-doped CNF (N-CNF) and nitrogen-doped CNC (N-CNC). We used Na_2_CO_3_ because it is water-soluble and can be removed from N-CNM through steps of water washing. We found that the Na_2_CO_3_ catalyst prepared by us is active and selective for mass formation of N-CNF and N-CNC. By means of CVD using Na_2_CO_3_ as catalyst, high-purity N-CNM can be obtained after washing the products with deionized water and ethanol. The approach is simple, inexpensive, and environment-benign, and can be used for mass production of high-purity N-CNF and N-CNC.

## Methods

All materials used were commercially available and analytically pure. In the present study, we employed Na_2_CO_3_ as catalyst. First, we mixed 10 g of Na_2_CO_3_ (in powder form) in 200 ml of deionized water at room temperature (RT) with continuous stirring. Once a transparent solution was obtained, the solution was kept at 80°C for several hours and allowed to cool down to RT for the precipitation of a white powder. The powder was filtered out, dried, and ground into tiny particles.

We placed 0.5 g of catalyst at the center of a ceramic boat with two open ends. The boat was then put inside a quartz tube with a thermocouple attached to its center. For the CVD reaction, we used acetylene as carbon source and ammonia as nitrogen source. After the reaction chamber was purged with argon for the elimination of oxygen, the sources were introduced into the system at either 450°C or 500°C at a C_2_H_2_/NH_3_ flow rate ratio of 1:1 for 6 h. To study the effect of changing the flow rate ratio, we also introduced acetylene and ammonia at a C_2_H_2_/NH_3_ flow rate ratio of 5:1 at 450°C for 6 h. After the reaction, argon was again introduced to protect the product from oxidation until the system was cooled down to RT. To remove the catalyst and to avoid organic outgrowth, the as-obtained products were repeatedly washed with deionized water and ethanol. Compared to the methods commonly used for CNM purification, the one used in the present study causes no damage to the desired product.

The morphologies of samples were examined using a transmission electron microscope (TEM) operated at an accelerating voltage of 200 kV and a field emission scanning electron microscope (FE-SEM) operated at an accelerating voltage of 5 kV. Fourier transform infrared (FTIR) spectroscopic studies of samples (in KBr pellets) were conducted over a Nocolet 510P spectrometer (Thermo Nocolet, Stanford, CT, USA). The surface analysis of products was carried out by means of X-ray photoelectron spectroscopy (XPS, PHI 5000 VersaProbe, UIVAC-PHI Inc., Chigasaki, Kanagawa, Japan). The products were examined on an X-ray powder diffractometer (XRD) at RT for phase identification using CuK_α_ radiation (model D/Max-RA, Rigaku Corporation, Tokyo, Japan). Raman spectroscopic investigations were performed over a Jobin-Yvon Labram HR800 instrument (Horiba, Ann Arbor, MI, USA) with 514.5-nm Ar laser excitation. The photoluminescence (PL) spectra were collected at RT over a spectrofluorophotometer (Shimadzu RF-5301 PC; Shimadzu Co. Ltd., Beijing, China) using a Xe lamp as light source. For PL investigation, about 0.1 mg of sample was ultrasonically dispersed in 5 ml of deionized water. Thermoanalysis was carried out using a thermal analysis system (NETZSCH STA 449C; NETZSCH Company, Shanghai, China) with the sample heated in air at a rate of 20°C/min.

## Results and discussion

We observed that when reaction temperature is higher than 500°C or lower than 400°C, the yield of CNM is small (TEM observation). Above 500°C, there is heavy decomposition of Na_2_CO_3_ into sodium oxide and CO_2_, a situation unfavorable for CNM formation. Below 400°C, the decomposition of acetylene becomes unfavorable. Since there could be Na_2_CO_3_ decomposition at certain reaction temperatures, we do not choose weight change as a means to measure product yields. Shown in Table 1 are the conditions used for the generation of CNM.

**Table 1:** Preparation summary of samples

**Reaction temperature (°C)**	**Flow rate ratios (C**_**2**_**H**_**2**_**/NH**_**3**_**)**	**Sample name**
450	C_2_H_2_ only	C450
450	5:1	C5N1
450	1:1	C450N
500	1:1	C500N

**Figure 1:**
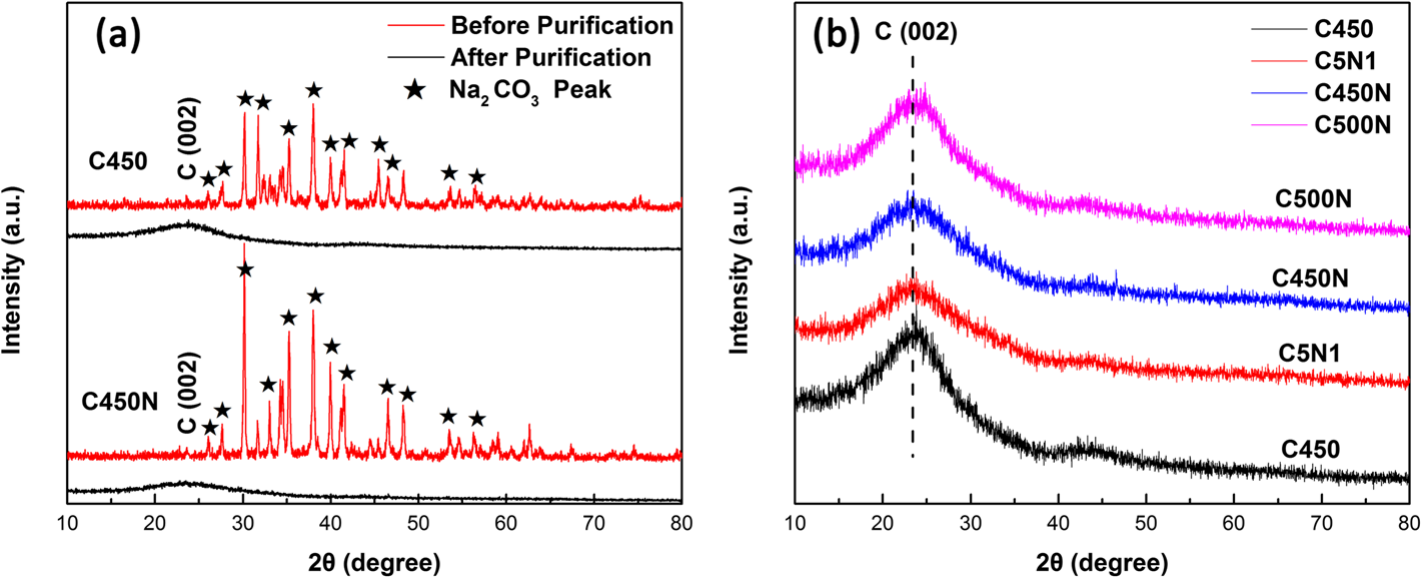
XRD patterns of (a) as-obtained and (b) purified samples.

Figure 1 shows the XRD patterns of the as-obtained and purified samples. The peaks of Na_2_CO_3_ can be indexed to the monoclinic phase of Na_2_CO_3_ (JCPDS 37–0451) with *a =* 8.906 Å, *b =* 5.238 Å, and *c* = 6.045 Å. Figure 1a,b is the patterns of C450 and C450N before and after purification, respectively. It is apparent that there are graphite carbon and Na_2_CO_3_ in CNM and N-CNM before purification. After repeated washing with water and ethanol, there is complete elimination of Na_2_CO_3_ as well as ethanol-soluble organic outgrowth. With the incorporation of nitrogen, there is decline of graphite signal intensity.

**Figure 2:**
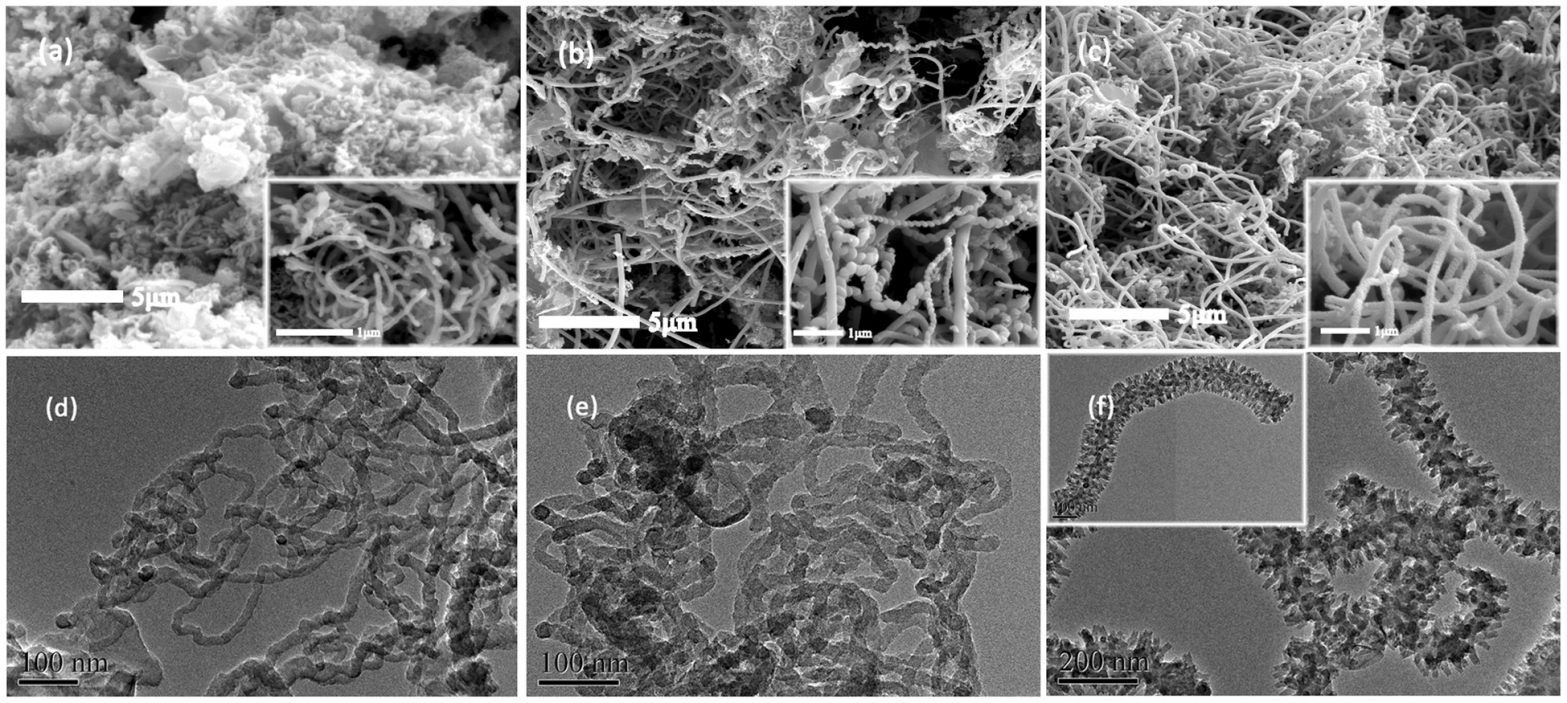
**FE-SEM and TEM images of C450, C450N, and C500N.** FE-SEM images of **(a)** C450, **(b)** C450N, and **(c)** C500N, and the TEM images of **(d)** C450, **(e)** C450N, and **(f)** C500N (insets are the corresponding high-magnification images).

Figure 2 shows the FE-SEM and TEM images of the purified samples. The selectivity to carbon species was determined statistically according to the number of counts of CNM at different regions of the TEM and FE-SEM images. The images of C5N1 are not given here for they are similar to those of C450 and C450N. As shown in Figure 2a,d, the major constitution of C450 is long and composed of linear carbon nanofibers (LCNF). The rest is irregular carbon complexes, and there is no detection of helical carbon nanofibers (HCNF). According to the TEM images, the average diameter of LCNF is *ca*. 20 nm. In other words, LCNF can be synthesized in large scale with high selectivity using this method. As shown in Figure 2b,e, the major product of C450N is still LCNF, but there is sighting of helical structures. As shown in the inset of Figure 2b, there are sightings of long HCNF. The TEM images indicate that the obtained LCNF and HCNF have average diameter of *ca*. 30 nm. The results show that with the doping of nitrogen into graphitic lattices, there is change in CNM morphology: the generation of helical structures. When the reaction temperature is 500°C, the major product of C500N is the long spiny carbon nanofibers (SCNF) (Figure 2c,f), having average diameter of *ca*. 100 nm. It is known that reaction temperature is a parameter that affects the synthesis of nanomaterials in terms of morphology, structure, and component. Through the control of morphology, structure, and/or component, it is possible to obtain CNM of particular properties. In the case of long SCNF, the material is enriched with multi-pillar structures and is relatively large in specific surface area. With such physical properties, the material can be used as support for better dispersion of nanoparticles.

XPS O1*s*, C1*s*, and N1*s* spectra were obtained for the determination of surface composition and bonding environment of C and N atoms of the purified samples. The nitrogen content of a particular product is defined as 100 N/(C + N + O) at.%. As depicted in Table 2, the amounts of nitrogen in C450, C5N1, C450N, and C500N are 0%, 1.77%, 2.86%, and 2.10%, respectively. It is noted that the oxygen contents of the four samples are about 4%. Based on the results, we deduce that a rise of nitrogen source at reaction temperature of 450°C results in products higher in nitrogen content. However, with a rise of reaction temperature from 450°C to 500°C, there is a slight decline of nitrogen content. It is plausible that NH_3_ decomposition is enhanced with temperature rise, but the concurrent decomposition of catalyst goes against the formation of nitrogen-doped CNT. That C500N is lower than C450N in nitrogen content is a net consequence of the two actions.

**Table 2:** Nitrogen content of samples

**Sample name**	**Nitrogen content (at.%)**
C450	0
C5N1	1.77
C450N	2.86
C500N	2.10

**Figure 3:**
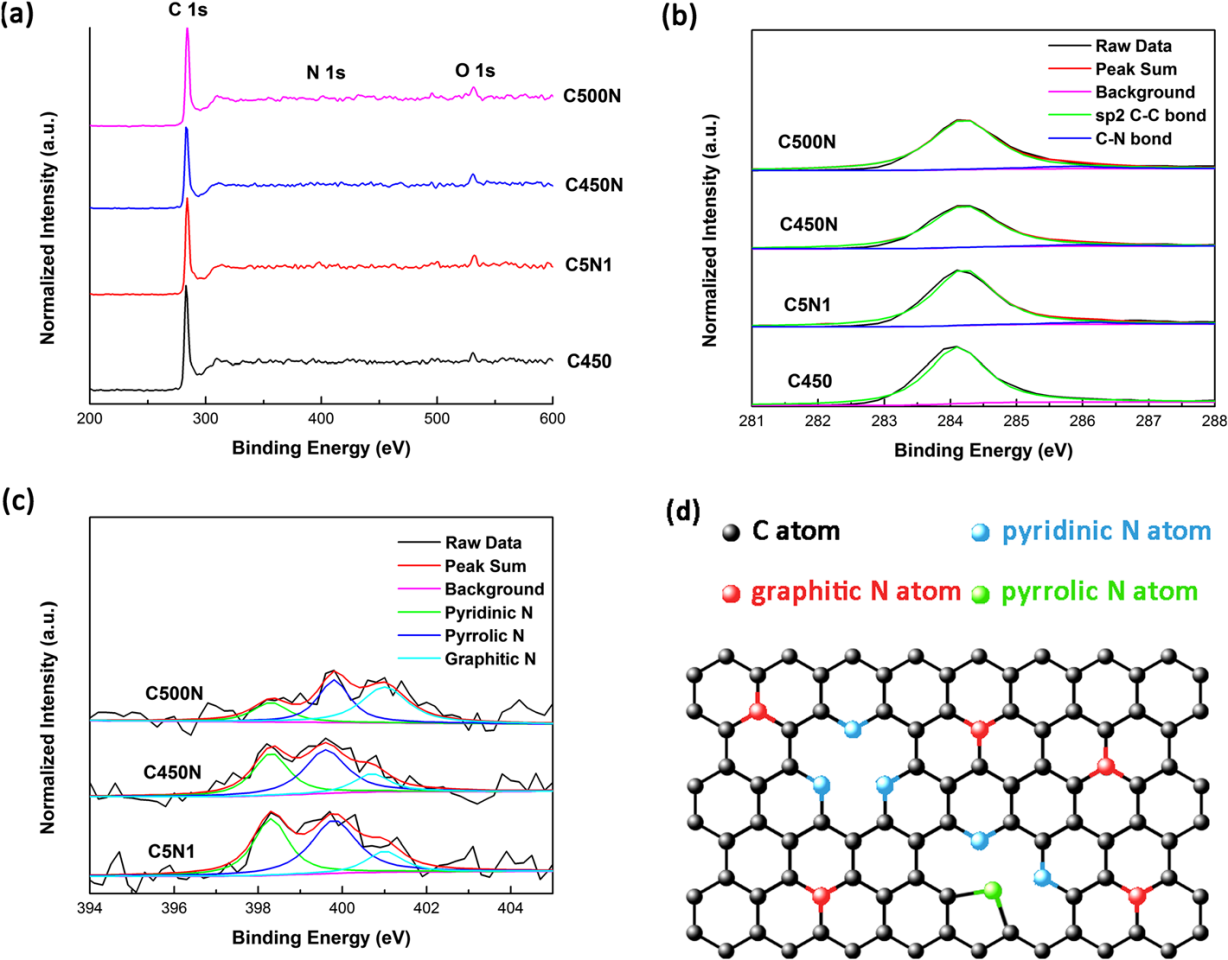
**XPS spectra of the purified samples. (a)** Survey scan, **(b)** C1*s* spectra, **(c)** N1*s* spectra, and **(d)** illustration of nitrogen configuration.

According to some researches, the electronic properties of CNM can be tuned by doping nitrogen atoms into the carbon lattices and be regulated by controlling the type, concentration, and content of dopants [56,57]. We observe that C450, C5N1, C450N, and C500N show C1*s*, N1*s*, and O1*s* peaks at around 284, 400, and 532 eV, respectively (Figure 3a). As shown in Figure 3b, the C1*s* peak can be deconvoluted into two components at 284.1 and 285.8 eV. The stronger one at 284.1 eV is ascribed to the carbon of *sp*^2^-hybridized C-C bonds whereas that at 285.8 eV to carbon of C-N bonds. There are three primary statuses of nitrogen configuration in nitrogen-doped CNMs: graphitic (substitutional nitrogen), pyridine-like, and pyrrole-like. In order to analyze the electronic state of nitrogen atoms in CNMs, we focused our attention especially to the N1*s* spectra, as revealed in Figure 3c. The peak around 398.3 eV is attributed to *sp*^3^-hybridized nitrogen of the tetrahedral phase; the nitrogen is pyridine-type and is connected with the defective graphite sheets. The peak at 399.8 eV is ascribable to nitrogen with a local structure alike that of pyrrole, and the nitrogen is hence considered as pyrrole-type. The peak at 401.0 eV corresponds to *sp*^2^-hybridized nitrogen of trigonal phase, and the nitrogen is graphite-type or substitutional type. The composition of the three types of nitrogen is reflected by the area ratio of the corresponding N1*s* peaks. With rise of reaction temperature from 400°C to 500°C, there is a significant increase of graphitic nitrogen relative to that of pyridine-type nitrogen. It is deduced that the formation of graphitic configuration becomes more favorable with the rise of temperature.

**Figure 4:**
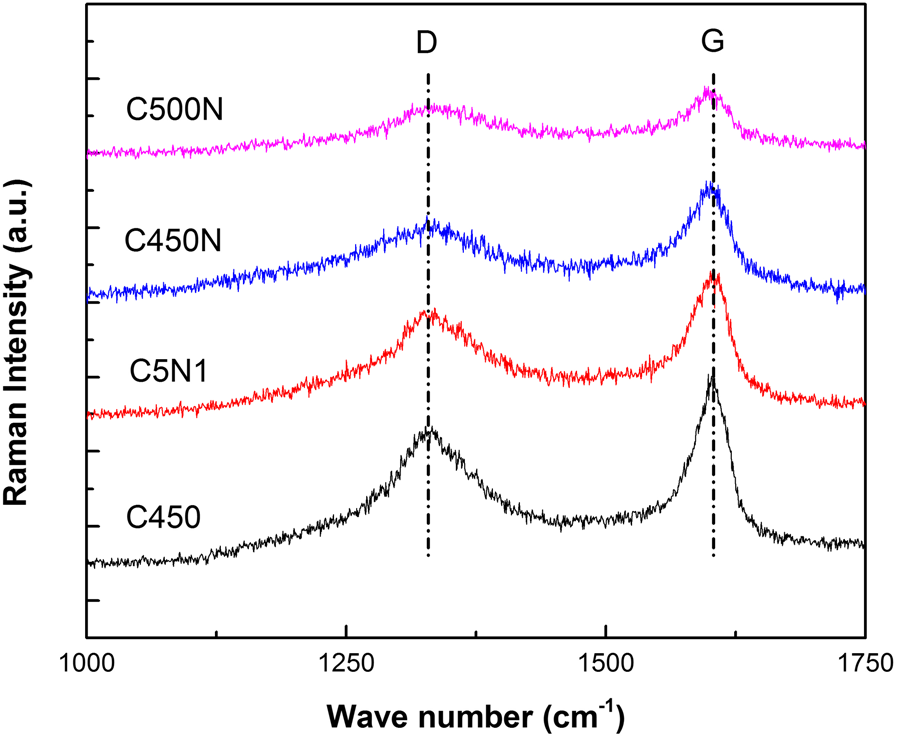
Raman spectra of C450, C5N1, C450N, and C500N.

Figure 4 shows the Raman spectra of C450, C5N1, C450N, and C500N. Each of the samples exhibits two peaks. The one at about 1,340 cm^-1^ (called D band) is associated with amorphous carbon relating to the vibration of carbon atoms with dangling bonds of disordered graphite. The peak at about 1,600 cm^-1^ (called G band) is related to the double-degenerate E2*g* mode of graphite, corresponding to the vibration of triple-degenerate *sp*^2^ hybrid bond. The intensity ratio of G band and D band (*I*_G_/*I*_D_) is generally used to identify the crystallinity of graphite. Lower *I*_G_/*I*_D_ means more defect or vacancy. The intensity ratios of C450, C5N1, C450N, and C500N are listed in Table 3.

**Table 3:** **The *I***_**G**_**/*I***_**D **_**intensity ratios of all samples**

**Sample name**	***I***_**G**_**/*I***_**D**_
C450	1.326
C5N1	1.287
C450N	1.255
C500N	1.239

Compared with C5N1, C450N is lower in *I*_G_/*I*_D_ value. The C_2_H_2_/NH_3_ flow rate ratio for the formation of C5N1 is 5:1 whereas that of C450N is 1:1. In other words, with a source flow richer in nitrogen, there is rise of nitrogen content, and with more defects or vacancies in N-CNM, there is decline of *I*_G_/*I*_D_ value. With the rise of reaction temperature from 450°C to 500°C, there is slight decrease of nitrogen content but enhanced formation of amorphous carbon, and the net result is the further decline of *I*_G_/*I*_D_ value.

**Figure 5:**
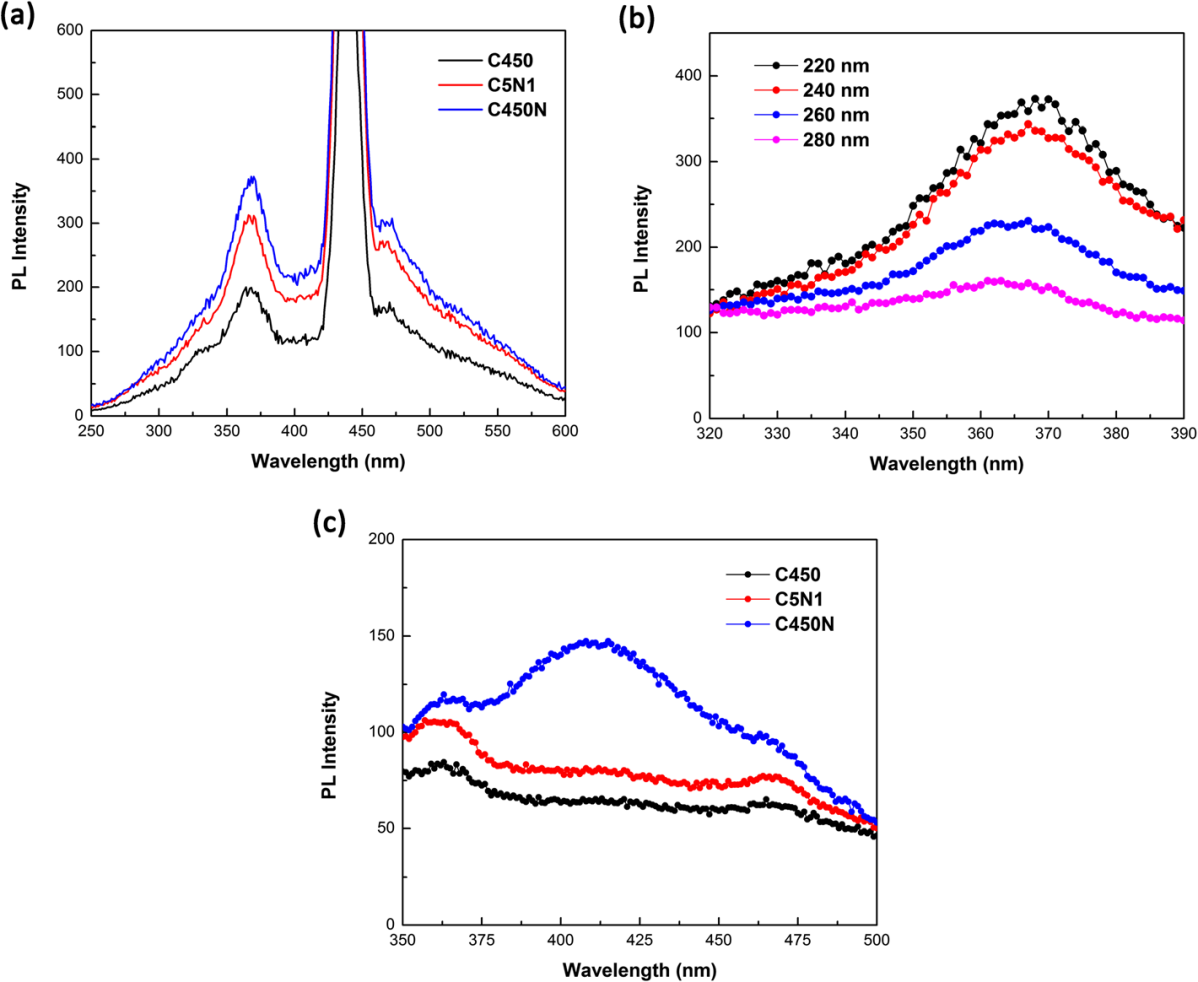
**PL spectra of C450, C5N1, and C450. (a)** C450, C5N1, and C450 with an excitation wavelength of 220 nm. **(b)** C450N with different excitation wavelengths ranging from 220 to 280 nm. **(c)** C450, C5N1, and C450 with an excitation wavelength of 300 nm.

The PL spectra of C450, C5N1, and C450N obtained with an excitation source of 220 nm wavelength are showed in Figure 5a. It is known that pristine CNM exhibits strong UV PL at 368 nm at RT. In the present study, we find that by doping of nitrogen into CNM, there is enhancement of UV PL, and PL signal intensifies with the rise of nitrogen content. The peak at 468 nm is a sideband peak, and its intensity is usually weaker than that of 368 nm. The super peak at about 440 nm is the double wavelength of 220 nm attributable to the excitation wavelength. In Figure 5b, with the excitation wavelength increasing from 220 to 280 nm, the intensity of the PL peak at 368 nm decreases. When the excitation wavelength reaches 300 nm, there is the detection of a peak at about 410 nm over the C450N sample as shown in Figure 5c. The peak is a purple band. There is no detection of such a peak at about 410 nm over the C450 and C5N1 samples. We ascribe the phenomenon to the impurity transition level induced by doping nitrogen of a certain concentration into the graphite lattice. It is hence possible to modulate the luminescence peak in a controllable manner from visible light to the UV band by doping CNT with different concentrations of nitrogen.

**Figure 6:**
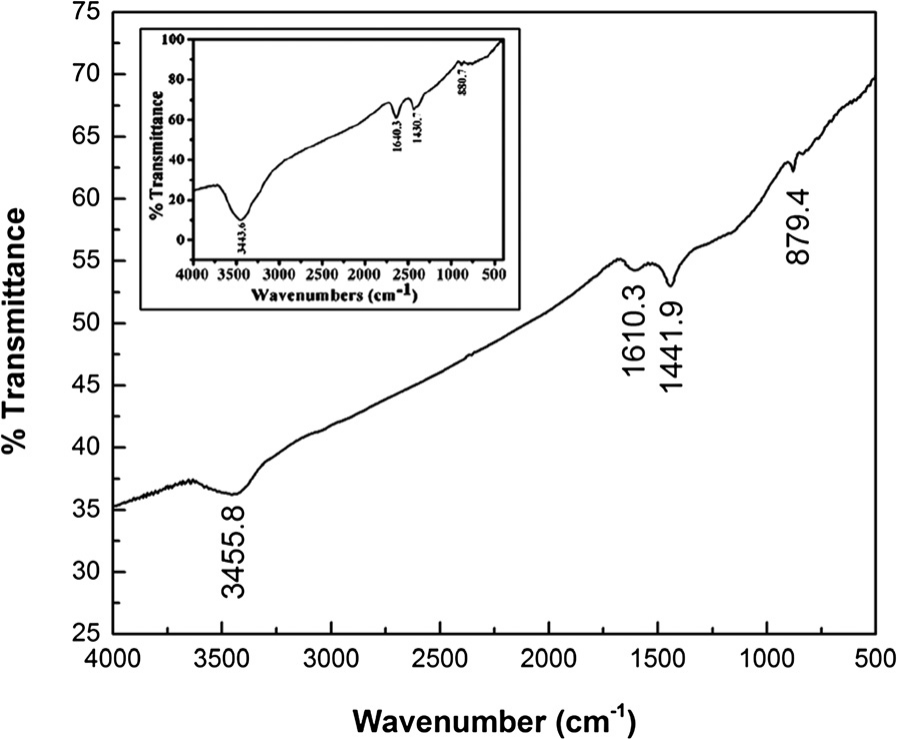
**FTIR spectrum of C450N.** Inset is the FTIR spectrum of C450, after [53].

Figure 6 is the FTIR spectrum of C450N. The peak at 3,455.8 cm^-1^ can be ascribed to the stretching vibration of unsaturated –CH = CH–. The peaks at 1,610.3 and 1,441.9 cm^-1^ are ascribed to –C-H stretching vibration while that at 879.4 cm^-1^ to –C-H deformation vibration. Compared to the FTIR result of our previous study [53], the nitrogen-doped CNM shows weaker peak intensity and poorer transmittance plausibly due to the presence of defects or vacancies.

**Figure 7:**
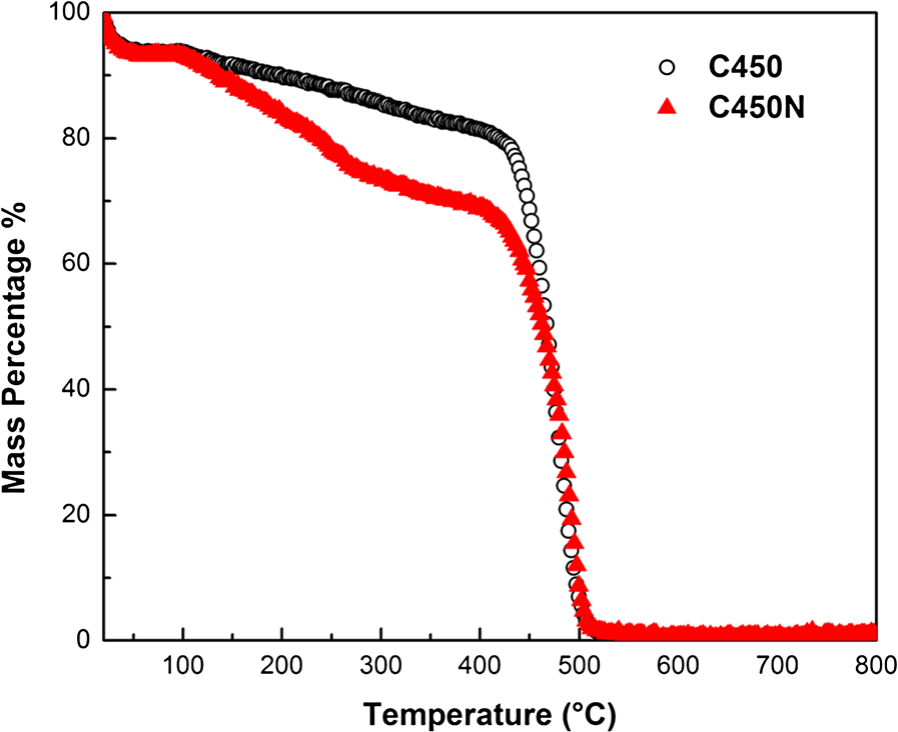
TGA curve of C450 and C450N.

We tested the oxidation resistance of C450 and C450N. As shown in Figure 7, both samples are sharply oxidized at about 460°C, at a temperature lower than that for the oxidation of CNM generated in CVD processes using iron-group metals or their alloys as catalysts [58,59]. Furthermore, the oxidation of C450N starts at about 460°C, and it is not so with C450. The results suggest that there are more active defects and amorphous carbon in C450N in comparison with C450.

## Conclusions

By controlling the acetylene decomposition temperature, N-CNF and N-CNC can be selectively synthesized in large scale over Na_2_CO_3_. Due to the water-soluble property of NaCO_3_, the products can be obtained in high purity through steps of water and ethanol washing. The CVD process using Na_2_CO_3_ as catalyst is simple, inexpensive, and environment-benign. We detect graphitic, pyridine-like as well as pyrrole-like N species in the nitrogen-doped CNM. Compared to the non-doped pristine CNM, the nitrogen-doped ones show enhanced UV PL intensity.

## Competing interests

The authors declare that they have no competing interests.

## Authors’ contributions

WZ and QD designed the study and guided this work. XYS, XJY, and XSQ participated in the design of the study. QD carried out the experiments, analyzed the data, and drafted the manuscript. WZ and CTA checked and revised the manuscript. CTA and YWD gave precious suggestions to this work. All authors read and approved the final manuscript.
